# Morphology and molecular genetics reveal two new *Leptobrachella* species in southern China (Anura, Megophryidae)

**DOI:** 10.3897/zookeys.776.22925

**Published:** 2018-07-26

**Authors:** Jian Wang, Jianhuan Yang, Yao Li, Zhitong Lyu, Zhaochi Zeng, Zuyao Liu, Youhua Ye, Yingyong Wang

**Affiliations:** 1 State Key Laboratory of Biocontrol / The Museum of Biology, School of Life Sciences, Sun Yat-sen University, Guangzhou 510275, PR China Sun Yat-sen University Guangzhou China; 2 Kadoorie Conservation China, Kadoorie Farm and Botanic Garden, Lam Kam Road, Tai Po, Hong Kong, PR China Kadoorie Farm and Botanic Garden Hong Kong Hong Kong; 3 Zhongkai University of Agriculture and Engineering, Guangzhou 510275, PR China Zhongkai University of Agriculture and Engineering Guangzhuo China

**Keywords:** China, *Leptobrachellayunkaiensis* sp. n., *L.wuhuangmontis* sp. n., morphology, phylogenetic, species diversity

## Abstract

Based on morphological and phylogenetic analyses (16S rRNA mtDNA), two new species of the genus *Leptobrachella* are described from southern China, namely *L.yunkaiensis* Wang, Li, Lyu & Wang, **sp. n.** from Dawuling Forest Station of Guangdong Province and *L.wuhuangmontis* Wang, Yang & Wang, **sp. n.** from Mt. Wuhuang of Guangxi Province. To date, the genus *Leptobrachella* contains 68 species, among which 13 species are known from China. The descriptions of the two new species further emphasize that the species diversity of the genus *Leptobrachella* from China is still highly underestimated and requires further investigations.

## Introduction

The genus *Leptolalax* Dubois, 1983 within the family Megophryidae Bonaparte, 1850 was currently found to be non-monophyletic with *Leptobrachella* Smith, 1925, and was assigned as a junior synonym of *Leptobrachella* based on a large-scale molecular analysis (Chen et al. 2018).Their results also rejected the hypothesis that *Leptolalax* consists of two subgenera as proposed by Delorme et al. (2006) and Dubois et al. (2010). At present, the genus *Leptobrachella* contains sixty-six species, widely distributed from southern China west to northeastern India and Myanmar, through mainland Indochina to peninsular Malaysia and the island of Borneo (Frost 2017; Nguyen et al. 2018; Rowley et al. 2016, 2017; Yang et al. 2016; Yuan et al. 2017). They are commonly known as Asian leaf litter frogs. Currently, eleven species of this genus are known from China, i.e., *L.alpinus* from Yunnan and Guangxi provinces, *L.laui* from southern Guangdong including Hong Kong, *L.liui* from Fujian, Jiangxi, Guangdong, Guangxi, Hunan and Guizhou provinces, *L.oshanensis* from Gansu, Sichuan, Chongqing, Guizhou and Hubei provinces, L.cf.pelodytoides (which may represents a undescribed taxon), *L.purpura*, *L.tengchongensis*, *L.ventripuntatus*, and *L.yingjiangensis* from Yunnan Province, and *L.sungi* and *L.maoershanensis* from Guangxi Province (Sung et al. 2014; Yang et al. 2016; Yuan et al. 2017; Yang et al. 2018).

**Figure 1.**
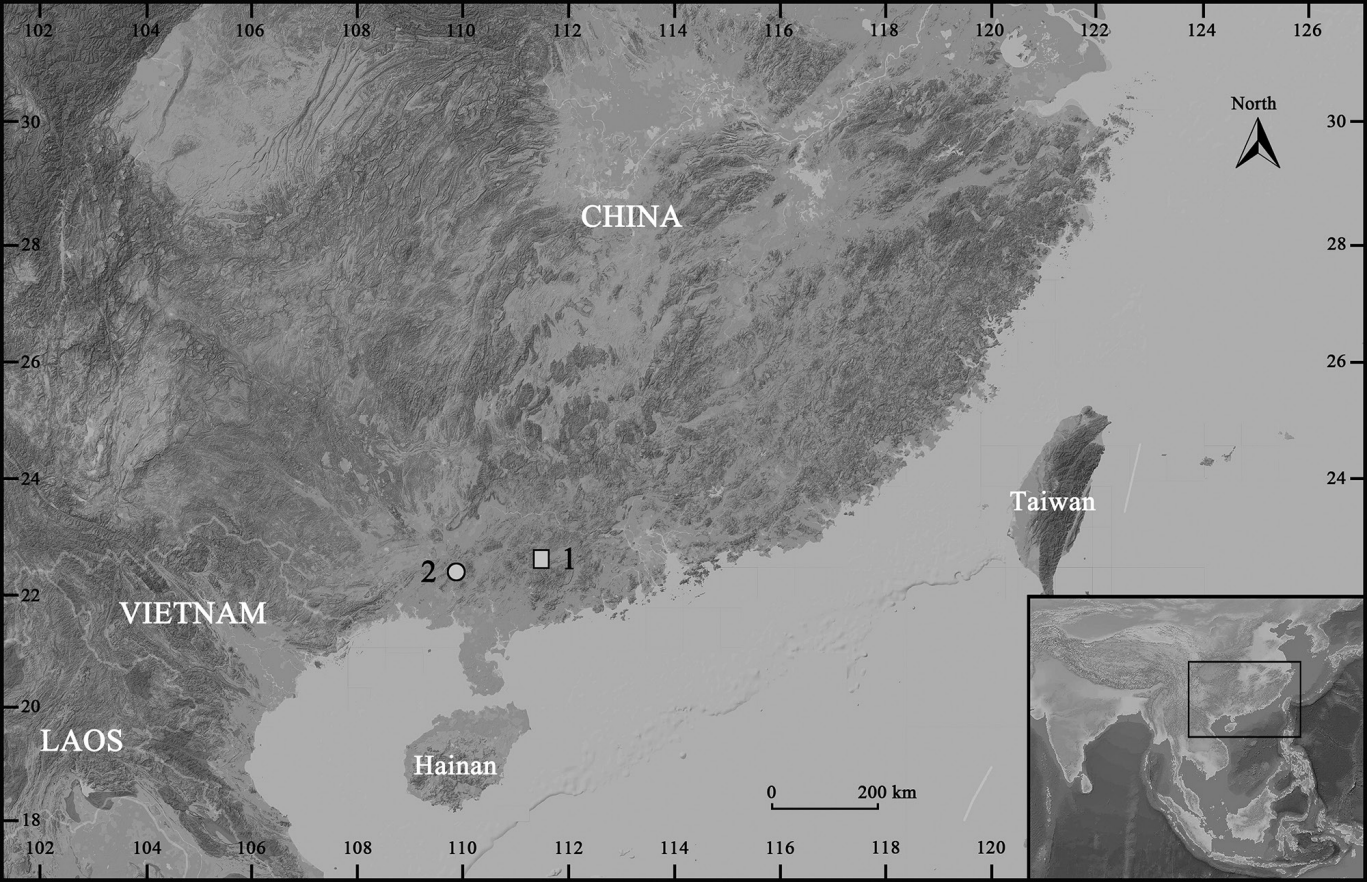
Collection localities of the two new *Leptobrachella* species: **1** the type locality of *Leptobrachellayunkaiensis* sp. n., Dawuling Forest Station in Guangdong Province **2** the type locality of *L.wuhuangmontis* sp. n., Mt. Wuhuang in Guangxi Province.

During field surveys in southern China from 2009 to 2016, a number of specimens were collected from Dawuling Forest Station of Guangdong Province and Mt. Wuhuang of Guangxi Province, respectively (Figure 1), that can all be morphologically assigned to the genus *Leptobrachella*, based on the following characters: (1) comparatively small size, snout-vent length no overlap than 60.0 mm, (2) rounded finger tips, the presence of an elevated inner palmar tubercle not continuous to the thumb, (3) presence of macroglands on body including supra-axillary, pectoral, femoral and ventrolateral glands, (4) vomerine teeth absent, (5) tubercles on eyelids present, and (6) anterior tip of snout with whitish vertical bar (Dubois 1983; Matsui 1997, 2006; Lathrop et al. 1998; Delorme et al. 2006; Das et al. 2010). Subsequent molecular studies on 16S rRNA mtDNA sequences revealed that this collection represents two different undescribed species which can be distinguished from each other and from all other recognized congeners by a combination of morphological characters and molecular divergences; they are described herein as two new species.

## Materials and methods

**Sampling.** For molecular analyses, a total of 65 samples (19 muscle tissues and 46 sequences downloaded from Genbank) from 29 species of the genus *Leptobrachella* were sequenced, in addition to two undescribed species from China, i.e., the population from Dawuling Forest Station of Guangdong Province and Mt. Wuhuang of Guangxi Province. Additionally, four sequences were downloaded from GenBank as the outgroups (see Table 1; *Pelobatessyriacus*, *Pelobatesvaraldii*, Leptobrachiumcf.chapaense and *Megophrysmajor*).

**Table 1.** Localities and voucher data for all specimens used in this study.

ID	Species	Locality	Voucher no.	GenBank No. 16S rRNA
**1**	*Leptobrachellayunkaiensis* sp. n.	China: Dawuling Forest Station, Maoming City, Guangdong	SYS a004663	MH605584
**2**	*Leptobrachellayunkaiensis* sp. n.	China: Dawuling Forest Station, Maoming City, Guangdong	SYS a004664 / CIB107272	MH605585
**3**	*Leptobrachellayunkaiensis* sp. n.	China: Dawuling Forest Station, Maoming City, Guangdong	SYS a004665	MH605586
**4**	*Leptobrachellayunkaiensis* sp. n.	China: Dawuling Forest Station, Maoming City, Guangdong	SYS a004666	MH605587
**5**	*Leptobrachellayunkaiensis* sp. n.	China: Dawuling Forest Station, Maoming City, Guangdong	SYS a004667	MH605588
**6**	*Leptobrachellayunkaiensis* sp. n.	China: Dawuling Forest Station, Maoming City, Guangdong	SYS a004668	MH605589
**7**	*Leptobrachellayunkaiensis* sp. n.	China: Dawuling Forest Station, Maoming City, Guangdong	SYS a004669	MH605590
**8**	*Leptobrachellayunkaiensis* sp. n.	China: Dawuling Forest Station, Maoming City, Guangdong	SYS a004690	MH605591
**9**	*Leptobrachellawuhuangmontis* sp. n.	China: Mt. Wuhuang, Pubei County, Guangxi	SYS a003485	MH605577
**10**	*Leptobrachellawuhuangmontis* sp. n.	China: Mt. Wuhuang, Pubei County, Guangxi	SYS a003486	MH605578
**11**	*Leptobrachellawuhuangmontis* sp. n.	China: Mt. Wuhuang, Pubei County, Guangxi	SYS a003487	MH605579
**12**	*Leptobrachellawuhuangmontis* sp. n.	China: Mt. Wuhuang, Pubei County, Guangxi	SYS a003499	MH605580
**13**	*Leptobrachellawuhuangmontis* sp. n.	China: Mt. Wuhuang, Pubei County, Guangxi	SYS a003500 / CIB107274	MH605581
**14**	*Leptobrachellawuhuangmontis* sp. n.	China: Mt. Wuhuang, Pubei County, Guangxi	SYS a003504	MH605582
**15**	* Leptobrachella aerea *	Vietnam: Quang Binh	RH60165	JN848437
**16**	* Leptobrachella applebyi *	Vietnam: Kon Tum	AMS R 173778	KR018108
**17**	* Leptobrachella applebyi *	Vietnam: Kon Tum	AMS R 173635	KU530189
**18**	* Leptobrachella bidoupensis *	Vietnam: Lam Dong	AMS R 173133	HQ902880
**19**	* Leptobrachella bidoupensis *	Vietnam: Lam Dong	NCSM 77321	HQ902883
**20**	* Leptobrachella bourreti *	Vietnam: Lao Cai	AMS R 177673	KR018124
**21**	* Leptobrachella eos *	Laos: Phongsaly	MNHN : 2004.0278	JN848450
**22**	* Leptobrachella firthi *	Vietnam: Kon Tum	AMS R 176524	JQ739206
**23**	* Leptobrachella fritinniens *	Malaysia: Borneo	KUHE55371	AB847557
**24**	* Leptobrachella gracilis *	Malaysia: Borneo	KUHE 55624	AB847560
**25**	* Leptobrachella hamidi *	Malaysia: Borneo	KUHE 17545	AB969286
**26**	* Leptobrachella heteropus *	Malaysia: Peninsula	KUHE 15487	AB530453
**27**	* Leptobrachella isos *	Vietnam: Gia Lai	VNMN A 2015.4 / AMS R 176480	KT824769
**28**	* Leptobrachella laui *	China: Tai Mo Shan, Hong Kong	SYS a002057	KM014546
**29**	* Leptobrachella laui *	China: San zhoutian, Shenzhen	SYSa002450	MH055904
**30**	* Leptobrachella laui *	China: Mt. Wutong, Shenzhen	SYS a003477	MH605576
**31**	* Leptobrachella liui *	China: Mt. Wuyi, Fujian	SYS a002478	MH605573
**32**	* Leptobrachella liui *	China: Mt. Wuyi, Fujian	SYS a002479	MH605574
**33**	* Leptobrachella liui *	China: Mt. Wuyi, Fujian	SYS a001597	KM014547
**34**	* Leptobrachella liui *	China: Mt. Tongbo, Jiangxi	SYS a001702	KM014548
**35**	* Leptobrachella liui *	China: Mt. Daiyun, Fujian	SYS a001736	KM014550
**36**	* Leptobrachella liui *	China: Dongkeng Town, Jingning County, Zhejiang	SYSa002732	MH605575
**37**	* Leptobrachella liui *	China: Dongkeng Town, Jingning County, Zhejiang	SYSa002733	MH055909
**38**	* Leptobrachella marmorata *	Malaysia: Borneo	KUHE 53227	AB969289
**39**	* Leptobrachella maura *	Malaysia: Borneo	SP 21450	AB847559
**40**	* Leptobrachella maoershanensis *	China: Maoershan, Guangxi	KIZ 019386	KY986931
**41**	* Leptobrachella melica *	Cambodia: Ratanakiri	MVZ 258198	HM133600
**42**	* Leptobrachella minima *	Thailand: Chiangmai	/	JN848369
**43**	* Leptobrachella nyx *	Vietnam: Ha Giang	AMNH A 163810	DQ283381
**44**	* Leptobrachella oshanensis *	China: Sichuan	SYS a001830	KM014810
**45**	* Leptobrachella pallida *	Vietnam: Lam Dong	UNS 00511	KU530190
**46**	* Leptobrachella picta *	Malaysia: Borneo	UNIMAS 8705	KJ831295
**47**	* Leptobrachella pluvialis *	Vietnam: Lao Cai	MNHN:1999.5675	JN848391
**48**	* Leptobrachella pyrrhops *	Vietnam: Lam Dong	ZMMU A-5208	KP017575
**49**	* Leptobrachella pyrrhops *	Vietnam: Lam Dong	ZMMU A-4873 (ABV-00213)	KP017576
**50**	* Leptobrachella sabahmontana *	Malaysia: Borneo	BORNEENSIS 12632	AB847551
**51**	* Leptobrachella rowleyae *	Vietnam: Da Nang City, Son Tra	ITBCZ 4113	MG682549
**52**	* Leptobrachella rowleyae *	Vietnam: Da Nang City, Son Tra	ITBCZ 4114	MG682550
**53**	* Leptobrachella rowleyae *	Vietnam: Da Nang City, Son Tra	ITBCZ 2790	MG682551
**54**	* Leptobrachella rowleyae *	Vietnam: Da Nang City, Son Tra	ITBCZ 2783	MG682552
**55**	* Leptobrachella tengchongensis *	China: Tengchong County, Yunnan	SYS a004596	KU589208
**56**	* Leptobrachella tengchongensis *	China: Tengchong County, Yunnan	SYS a004598	KU589209
**57**	* Leptobrachella tengchongensis *	China: Tengchong County, Yunnan	SYS a004600	KU589210
**58**	* Leptobrachella ventripunctata *	Laos: Phongsaly	MNHN 2005.0116	JN848410
**59**	* Leptobrachella ventripunctata *	China: Zhushihe, Xishuangbanna, Yunnan	SYS a001768	KM014811
**60**	* Leptobrachella ventripunctata *	China: Zhushihe, Xishuangbanna, Yunnan	SYS a003957	MH605583
**61**	* Leptobrachella zhangyapingi *	Thailand: Chiang Mai	KJ-2013	JX069979
**62**	Leptobrachiumcf.chapaense	Vietnam: Lao Cai	AMS R 171623	KR018126
**63**	*Pelobatessyriac*us	/	MVZ 234658	AY236807
**64**	* Pelobates varaldii *	/	/	AY236808
**65**	* Megophrys major *	Vietnam: Kon Tum	AMS R 173870	KY476333

All specimens were previous to fixation in 10% buffered formalin and later transferred to 70% ethanol for preservation, and deposited at the Museum of Biology, Sun Yat-sen University (**SYS**) and Chengdu Institute of Biology, the Chinese Academy of Sciences (**CIB**), China; tissue samples were preserved in 95% ethanol for molecular studies.

**DNA Extraction, PCR and sequencing.** DNA was extracted from muscle tissue using a DNA extraction kit from Tiangen Biotech (Beijing) Co., Ltd. The mitochondrial gene 16S ribosomal RNA gene (16S rRNA) from each sample was sequenced. Fragments of the genes were ampliﬁed using primer pairs L3975 (5’-CGCCTGTTTACCAAAAACAT-3’) and H4551 (5’-CCGGTCTGAACTCAGATCACGT-3’) for 16S rRNA (Simon et al. 1994). PCR ampliﬁcations were performed in a 20 μl reaction volume with the following cycling conditions: an initial denaturing step at 95 °C for five min; 35 cycles of denaturing at 95 °C for 40 s, annealing at 53 °C for 40 s and extending at 72 °C for one min, and a ﬁnal extending step of 72 °C for 10 min. PCR products were puriﬁed with spin columns. The purified products were sequenced with both forward and reverse primers using BigDye Terminator Cycle Sequencing Kit according to the guidelines of the manufacturer. The products were sequenced on an ABI Prism 3730 automated DNA sequencer in Shanghai Majorbio Bio-pharm Technology Co., Ltd. All sequences have been deposited in GenBank (Table 1).

**Phylogenetic analyses.** Sequence alignments were first conducted using Clustal X 2.0 (Thompson et al. 1997), with default parameters and the alignment being checked and manually revised, if necessary. Tested in Jmodeltest v2.1.2 (Darriba et al. 2012) with Akaike and Akaike information criteria, the best-fitting nucleotide substitution models are GTR + I + G. Phylogenetic trees were analyzed using maximum likelihood (ML) implemented in RaxmlGUI 1.3 (Silvestro and Michalak 2012), and Bayesian inference (BI) using MrBayes 3.2.4 (Ronquist et al. 2012). For ML analysis, the maximum likelihood tree inferred from 1000 replicates was used to represent the evolutionary history of the taxa analyzed. Branches corresponding to partitions reproduced in less than 60% of bootstrap replicates were collapsed. For BI analysis, two independent runs with four Markov Chain Monte Carlo simulations were performed for ten million iterations and sampled every 1000^th^ iteration. The first 25% of samples were discarded as burn-in. Convergence of the markov Chain monte carlo simulations was assessed with PSRF ≤ 0.01 and ESS (effective sample size) value > 200 using Tracer v.1.4 (http://tree.bio.ed.ac.uk/software/tracer/). We also calculated pairwise sequence divergence based on uncorrected *p*-distance using MEGA 6.06 (Tamura et al. 2013).

**Morphometrics.** Measurements followed Fei et al. (2009) and Rowley et al. (2013), and were taken with digital calipers to the nearest 0.1 mm. These measurements were as follows:

**SVL** snout-vent length (from tip of snout to vent);

**HDL** head length (from tip of snout to rear of jaws);

**HDW** head width (head width of commissure of jaws);

**SNT** snout length (from tip of snout to anterior corner of eye);

**EYE** eye diameter (diameter of exposed portion of eyeball);

**IOD** interorbital distance (minimum distance between upper eyelids);

**INDY** internasal distance (distance between nares);

**TMP** tympanum diameter (horizontal diameter of tympanum);

**TEY** tympanum–eye distance (distance from anterior edge of tympanum to posterior corner of eye);

**TIB** tibia length (distance from knee to heel);

**ML** manus length (distance from tip of third digit to proximal edge of inner palmar tubercle);

**LAHL** length of lower arm and hand (distance from tip of the third finger to elbow);

**PL** pes length (distance from tip of fourth toe to proximal edge of the inner metatarsal tubercle);

**HLL** hindlimb length (distance from tip of fourth toe to vent).

Sex was determined by direct observation of calls in life, the presence of internal vocal sac openings, and the presence of eggs in abdomen through external inspection. Comparative morphological data of *Leptobrachella* species were obtained from examination of museum specimens (see Appendix 1) and from the references listed in Table 2. Due to the high likelihood of undiagnosed diversity within the genus (Rowley et al. 2016; Yang et al. 2016), where available, we relied on examination of topotypic material and/or original species descriptions.

**Table 2.** Obtained references of 66 known congeners of the genus *Leptobrachella*, respectively.

ID	*Leptobrachella* species	Literature obtained
**1**	*L.aereus* (Rowley, Stuart, Richards, Phimmachak & Sivongxay, 2010)	Rowley et al. 2010c
**2**	*L.alpinus* (Fei, Ye & Li, 1990)	Fei et al. 2009
**3**	*L.applebyi* (Rowley & Cao, 2009)	Rowley and Cao 2009
**4**	*L.arayai* (Matsui, 1997)	Matsui 1997
**5**	*L.ardens* (Rowley, Tran, Le, Dau, Peloso, Nguyen, Hoang, Nguyen & Ziegler, 2016)	Rowley et al. 2016
**6**	*L.baluensis* Smith, 1931	Dring 1983; Eto et al. 2016
**7**	*L.bidoupensis* (Rowley, Le, Tran & Hoang, 2011)	Rowley et al. 2011
**8**	*L.botsfordi* (Rowley, Dau, & Nguyen, 2013)	Rowley et al. 2013
**9**	*L.bourreti* (Dubois, 1983)	Ohler et al. 2011
**10**	*L.brevicrus* Dring, 1983	Dring 1983; Eto et al. 2015
**11**	*L.crocea* (Rowley, Hoang, Le, Dau & Cao, 2010)	Rowley et al. 2010a
**12**	*L.dringi* (Dubois, 1987)	Inger et al. 1995; Matsui and Dehling 2012
**13**	*L.eos* (Ohler, Wollenberg, Grosjean, Hendrix, Vences, Ziegler & Dubois, 2011)	Ohler et al. 2011
**14**	*L.firthi* (Rowley, Hoang, Dau, Le & Cao, 2012)	Rowley et al. 2012
**15**	*L.fritinniens* (Dehling & Matsui, 2013)	Dehling and Matsui 2013
**16**	*L.fuliginosa* (Matsui, 2006)	Matsui 2006
**17**	*L.gracilis* (Günther, 1872)	Günther 1872; Dehling 2012b
**18**	*L.hamidi* (Matsui, 1997)	Matsui 1997
**19**	*L.heteropus* (Boulenger, 1900)	Boulenger 1900
**20**	*L.isos* (Rowley, Stuart, Neang, Hoang, Dau, Nguyen & Emmett, 2015)	Rowley et al. 2015a
**21**	*L.itiokai* Eto, Matsui & Nishikawa, 2016	Eto et al. 2016
**22**	*L.juliandringi* Eto, Matsui & Nishikawa, 2015	Eto et al. 2015
**23**	*L.kajangensis* (Grismer, Grismer & Youmans, 2004)	Grismer et al. 2004
**24**	*L.kalonensis* (Rowley, Tran, Le, Dau, Peloso, Nguyen, Hoang, Nguyen & Ziegler, 2016)	Rowley et al. 2016
**25**	*L.kecil* (Matsui, Belabut, Ahmad & Yong, 2009)	Matsui et al. 2009
**26**	*L.khasiorum* (Das, Tron, Rangad & Hooroo, 2010)	Das et al. 2010
**27**	*L.lateralis* (Anderson, 1871)	Anderson 1871; Humtsoe et al. 2008
**28**	*L.laui* (Sung, Yang & Wang, 2014)	Sung et al. 2014
**29**	*L.liui* (Fei & Ye, 1990)	Fei et al. 2009; Sung et al. 2014
**30**	*L.macrops* (Duong, Do, Ngo, Nguyen & Poyarkov, 2018)	Duong et al. 2018
**31**	*L.maculosa* (Rowley, Tran, Le, Dau, Peloso, Nguyen, Hoang, Nguyen & Ziegler, 2016)	Rowley et al. 2016
**32**	*L.maoershanensis* (Yuan, Sun, Chen, Rowley & Che, 2017)	Yuan et al. 2017
**33**	*L.marmorata* (Matsui, Zainudin & Nishikawa, 2014)	Matsui et al. 2014b
**34**	*L.maura* (Inger, Lakim, Biun & Yambun, 1997)	Inger et al. 1997
**35**	*L.melanoleuca* (Matsui, 2006)	Matsui 2006
**36**	*L.melica* (Rowley, Stuart, Neang & Emmett, 2010)	Rowley et al. 2010b
**37**	*L.minima* (Taylor, 1962)	Taylor 1962; Ohler et al. 2011
**38**	*L.mjobergi* Smith, 1925	Eto et al. 2015
**39**	*L.nahangensis* (Lathrop, Murphy, Orlov & Ho, 1998)	Lathrop et al. 1998
**40**	*L.natunae* (Günther, 1895)	Günther 1895
**41**	*L.nokrekensis* (Mathew & Sen, 2010)	Mathew and Sen 2010
**42**	*L.nyx* (Ohler, Wollenberg, Grosjean, Hendrix, Vences, Ziegler & Dubois, 2011)	Ohler et al. 2011
**43**	*L.oshanensis* (Liu, 1950)	Fei et al. 2009
**44**	*L.pallida* (Rowley, Tran, Le, Dau, Peloso, Nguyen, Hoang, Nguyen & Ziegler, 2016)	Rowley et al. 2016
**45**	*L.palmata* Inger & Stuebing, 1992	Inger and Stuebing 1992
**46**	*L.parva* Dring, 1983	Dring 1983
**47**	*L.pelodytoides* (Boulenger, 1893)	Boulenger 1893; Ohler et al. 2011
**48**	*L.petrops* (Rowley, Dau, Hoang, Le, Cutajar & Nguyen, 2017)	Rowley et al. 2017
**49**	*L.pictua* (Malkmus, 1992)	Malkmus 1992
**50**	*L.platycephala* (Dehling, 2012)	Dehling 2012a
**51**	*L.pluvialis* (Ohler, Marquis, Swan & Grosjean, 2000)	Ohler et al. 2000, 2011
**52**	*L.puhoatensis* (Rowley, Dau & Cao, 2017)	Rowley et al. 2016
**53**	*L.purpura* (Yang, Zeng & Wang, 2018)	Yang et al. 2018
**54**	*L.pyrrhops* (Poyarkov, Rowley, Gogoleva, Vassilieva, Galoyan & Orlov, 2015)	Poyarkov et al. 2015
**55**	*L.rowleyae* (Nguyen, Poyarkov, Le, Vo, Ninh, Duong, Murphy & Sang, 2018)	Nguyen et al. 2018
**56**	*L.sabahmontana* (Matsui, Nishikawa & Yambun, 2014)	Matsui et al. 2014a
**57**	*L.serasanae* Dring, 1983	Dring 1983
**58**	*L.sola* (Matsui, 2006)	Matsui 2006
**59**	*L.sungi* (Lathrop, Murphy, Orlov & Ho, 1998)	Lathrop et al. 1998
**60**	*L.tadungensis* (Rowley, Tran, Le, Dau, Peloso, Nguyen, Hoang, Nguyen & Ziegler, 2016)	Rowley et al. 2016
**61**	*L.tamdil* (Sengupta, Sailo, Lalremsanga, Das & Das, 2010)	Sengupta et al. 2010
**62**	*L.tengchongensis* (Yang, Wang, Chen & Rao, 2016)	Yang et al. 2016
**63**	*L.tuberosa* (Inger, Orlov & Darevsky, 1999)	Inger et al. 1999
**64**	*L.ventripunctata* (Fei, Ye & Li, 1990)	Fei et al. 2009
**65**	*L.yingjiangensis* (Yang, Zeng & Wang)	Yang et al. 2018
**66**	*L.zhangyapingi* (Jiang, Yan, Suwannapoom, Chomdej & Che, 2013)	Jiang et al. 2013

## Results

**Figure 2.**
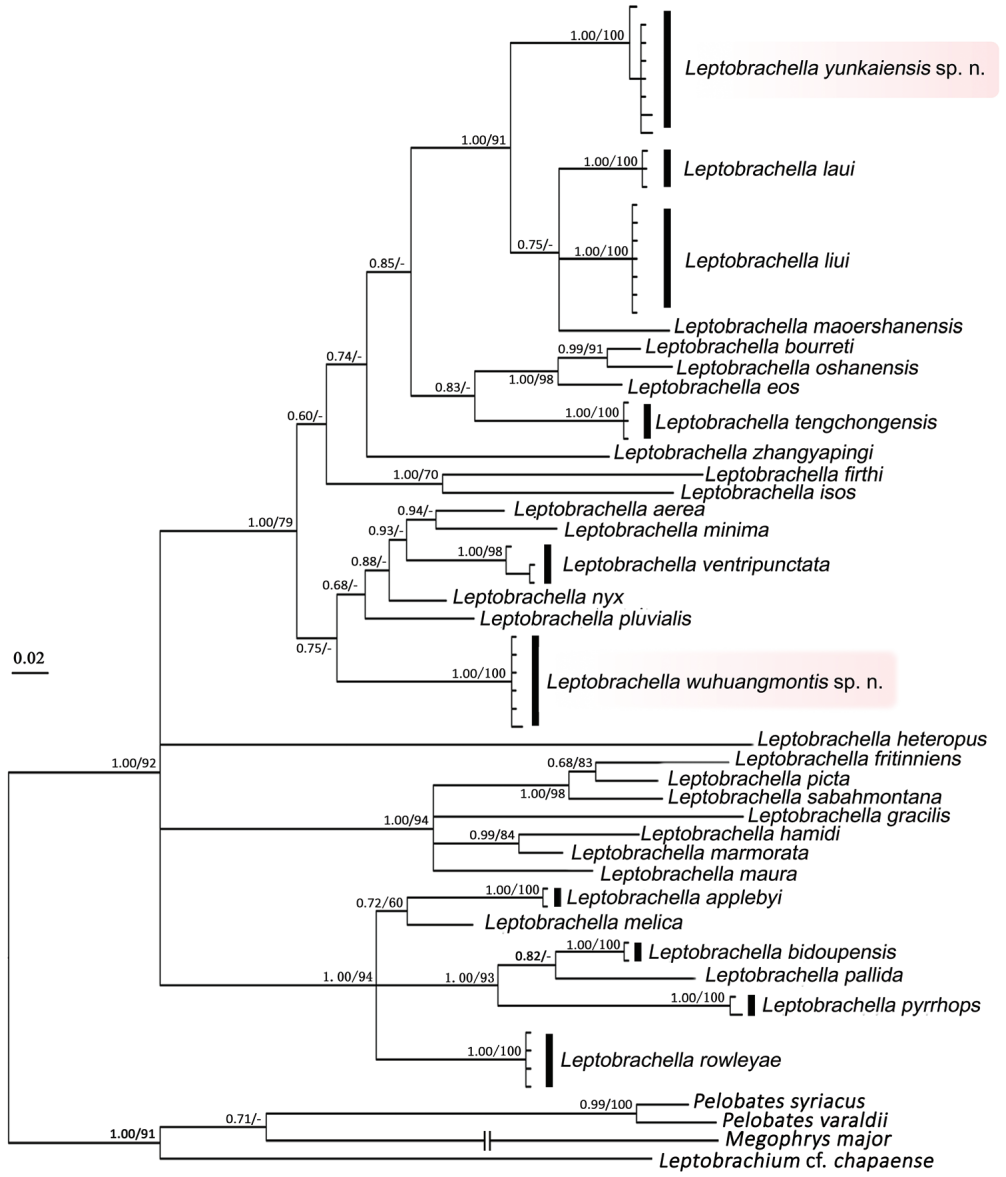
Bayesian inference tree derived from partial DNA sequences of the mitochondrial 16S r RNA gene. Numbers before slashes indicate Bayesian posterior probabilities (>60 retained) and numbers after slashes are bootstrap support for maximum likelihood (1000 replicates) analyses (>60 retained). The symbol “-” represents bootstrap value below 0.60/60%.

Bayesian inference (BI) and Maximum likelihood (ML) phylogenetic tree were constructed based on DNA sequences of the mitochondrial 16S gene with a total length of 476 bp. The two analyses resulted in essentially identical topologies (Figure 2) with clustered the population of *Leptobrachella* from Dawuling Forest Station with *L.laui*, *L.liui*, and *L.maoershanensis* with very high node supporting values (1.00 in BI and 91% in ML) and represented a separately evolving lineage. Besides, the population from Mt. Wuhuang was a distinct separately evolving lineage with high node supporting values (1.00/100% in BI and ML). The smallest pairwise genetic divergences between the population from Dawuling Forest Station and all other species of the genus *Leptobrachella* for which comparable sequences were included was 6.0–6.7% (with *L.liui*), and between population from Mt. Wuhuang and all other species was 7.4% (with *L.aerea*) (Table 3). These values were significantly larger than observed pairwise genetic distances between recognized species (*p*-distance = 2.6%, between *L.bourreti* and *L.oshanensis*). Given that the two populations both can be morphologically distinguished with each other, and from all known congeners, we herein describe these specimens as two new species, respectively.

**Table 3.** Uncorrected *p*-distances among *L.* species and outgroups based on 16S rRNA fragment (To be continued).

NO.	Species	1–8	9–14	15	16–17	18–19	20	21	22	23	24	25	26	27	28–30	31–37	38	39	40
**1–8**	*Leptobrachellayunkaiensis* sp. n.	0–0.3																	
**9–14**	*Leptobrachellawuhuangmontis* sp. n.	11.1–12.3	0–0.3																
**15**	* Leptobrachella aerea *	10.7–11.5	7.4	0															
**16–17**	* Leptobrachella applebyi *	15.4–15.9	13.8–14.2	14.5	0														
**18–19**	* Leptobrachella bidoupensis *	15.6–16.0	13.4–13.5	15.4	9.6	0													
**20**	* Leptobrachella bourreti *	8.1–8.9	10.3–10.7	10.3	14.3	17.2	0												
**21**	* Leptobrachella eos *	8.1–8.9	11.1–11.5	11.4	14.7	16.0	3.9	0											
**22**	* Leptobrachella firthi *	14.1–14.6	13.3–13.7	12.2	16.8	18.3	12.6	13.8	0										
**23**	* Leptobrachella fritinniens *	18.2–18.6	15.9–16.3	15.1	17.7	14.5	17.3	17.0	17.6	0									
**24**	* Leptobrachella gracilis *	20.3–20.8	19.9–20.4	18.1	16.4	18.7	19.5	20.8	22.2	13.0	0								
**25**	* Leptobrachella hamidi *	17.9–18.3	15.4–15.8	15.3	12.7	15.6	16.2	14.3	17.9	9.3	10.7	0							
**26**	* Leptobrachella heteropus *	20.1–21.0	16.6–17.7	17.5	15.5	17.4	20.5	21.4	22.4	19.6	20.8	17.3	0						
**27**	* Leptobrachella isos *	12.7–13.1	11.8–12.2	12.1	14.3	13.9	10.4	12.3	12.1	17.3	20.1	14.6	19.6	0					
**28–30**	* Leptobrachella laui *	6.3–6.7	12.5–12.9	10.7	16.1	17.9	8.8	8.8	13.4	18.0	19.4	15.8	20.8	13.3	0				
**31–37**	* Leptobrachella liui *	6.0–6.7	9.6	8.9	14.6	14.3	8.1	8.1	12.6	17.3	22.4	16.2	19.2	12.2	5.6	0			
**38**	* Leptobrachella marmorata *	16.7–16.8	13.9–14.3	14.5	10.9	15.7	14.7	14.3	15.6	9.6	11.4	4.2	18.2	14.7	16.2	15.1	0		
**39**	* Leptobrachella maura *	17.5–17.9	15.8	14.5	12.6	15.5	15.8	15.8	18.0	11.1	11.5	8.8	18.6	14.7	17.4	17.0	8.4	0	
**40**	* Leptobrachella maoershanensis *	6.7–7.1	10.0	18.5	15.1	14.8	9.9	9.9	16.6	18.5	21.2	16.3	19.2	13.4	6.7	5.7	16.3	17.5	0
**41**	* Leptobrachella melica *	16.6–17.0	13.0	12.6	5.6	9.1	14.3	15.4	17.1	16.1	15.2	12.3	16.0	15.3	16.5	15.7	12.4	13.1	15.8
**42**	* Leptobrachella minima *	11.1–11.9	10.8–11.2	6.3	15.0	16.0	11.1	11.9	12.8	17.8	20.0	16.6	18.6	13.3	8.9	8.2	15.8	16.6	9.2
**43**	* Leptobrachella nyx *	9.3–10.0	7.7–8.1	4.9	13.7	14.3	9.2	10.0	10.8	15.6	19.8	15.0	16.6	12.2	8.9	7.1	14.3	15.4	8.9
**44**	* Leptobrachella oshanensis *	8.5–9.2	11.1–11.5	10.7	15.1	18.1	2.6	5.0	12.6	17.3	19.5	16.7	22.2	11.2	8.1	8.5	16.3	16.3	11.1
**45**	* Leptobrachella pallida *	16.0–16.5	14.7–15.1	15.8	10.4	5.3	17.6	15.6	18.8	14.4	16.9	14.8	19.0	16.6	16.2	15.2	14.5	14.7	15.6
**46**	* Leptobrachella picta *	18.4–18.9	16.9–17.4	15.8	14.5	15.7	17.1	17.2	15.9	6.0	11.9	10.3	19.1	17.6	17.1	16.4	8.9	10.7	17.6
**47**	* Leptobrachella pluvialis *	8.2–8.6	8.2–8.5	6.4	13.9	14.8	10.3	11.1	13.5	16.6	19.2	16.7	16.0	14.2	8.9	7.9	15.2	16.7	6.8
**48–49**	* Leptobrachella pyrrhops *	14.3–15.5	13.1–14.0	13.5–13.9	12.3–12.7	9.0–9.3	16.7–17.1	16.0–16.5	17.2–17.6	15.8–16.2	17.4–17.8	16.1–16.5	17.0–17.4	14.3–14.7	15.5–15.9	15.2–15.6	15.8–16.2	17.2–17.6	14.4–14.8
**50**	* Leptobrachella sabahmontana *	17.9–18.4	15.4–15.9	15.4	12.9	15.0	15.8	16.0	15.9	7.0	12.7	10.0	21.3	16.3	17.0	16.3	8.5	8.9	17.9
**51–53**	* Leptobrachella tengchongensis *	11.1–11.9	12.2–12.6	8.5	15.3	15.8	8.1	7.8	11.2	16.1	21.2	14.2	19.1	9.3	8.1	8.5	14.7	15.4	10.3
**54–56**	* Leptobrachella ventripunctata *	11.5–12.7	8.5–10.0	6.7–7.7	16.3–16.6	17.5–18.0	11.1–12.3	11.9–13.1	11.1–11.5	15.6–16.5	20.4–21.2	14.7–15.1	18.5	11.5–11.8	10.4–11.6	9.0–10.1	14.0	15.0–15.8	10.0–10.4
**57**	* Leptobrachella zhangyapingi *	12.5–12.9	13.3	10.6	15.4	16.2	11.0	10.3	13.5	18.9	22.4	18.3	20.5	12.0	10.3	10.6	16.8	16.8	12.1
**58–61**	* Leptobrachella rowleyae *	16.3–16.7	13.5–14.0	14.3	7.8	10.7	15.1	16.3	17.3	16.8	19.1	14.1	17.8	16.3	15.8	15.1	13.4	14.8	15.1
**62**	Leptobrachium cf. chapaense	25.3–25.7	23.8–24.3	25.5	23.7	27.5	28.4	28.8	29.3	27.4	27.0	24.8	25.7	25.6	26.8	25.6	23.7	22.2	25.2
**63**	*Pelobatessyriacu*s	26.6–27.6	24.4–24.9	26.2	23.2	25.0	26.7	27.2	27.2	22.3	22.6	24.0	26.0	29.4	26.1	25.8	22.4	22.6	26.8
**64**	* Pelobates varaldii *	27.0–28.0	25.1–25.6	25.3	23.1	25.4	25.7	25.4	27.5	22.4	23.9	24.1	27.4	28.9	25.7	25.4	21.6	24.3	27.2
**65**	* Megophrys major *	28.8–29.9	26.4–27.0	27.6	25.6	28.8	25.8	26.2	30.9	28.1	25.9	25.1	27.3	27.4	29.4	27.6	25.2	23.1	28.2

**Table 3.** Continued.

NO.	Species	41	42	43	44	45	46	47	48–49	50	51–53	54–56	57	58–61	62	63	64	65
**1–8**	*Leptobrachellayunkaiensis* sp. n.																	
**9–14**	*Leptobrachellawuhuangmontis* sp. n.																	
**15**	* Leptobrachella aerea *																	
**16–17**	* Leptobrachella applebyi *																	
**18–19**	* Leptobrachella bidoupensis *																	
**20**	* Leptobrachella bourreti *																	
**21**	* Leptobrachella eos *																	
**22**	* Leptobrachella firthi *																	
**23**	* Leptobrachella fritinniens *																	
**24**	* Leptobrachella gracilis *																	
**25**	* Leptobrachella hamidi *																	
**26**	* Leptobrachella heteropus *																	
**27**	* Leptobrachella isos *																	
**28–30**	* Leptobrachella laui *																	
**31–37**	* Leptobrachella liui *																	
**38**	* Leptobrachella marmorata *																	
**39**	* Leptobrachella maura *																	
**40**	* Leptobrachella maoershanensis *																	
**41**	* Leptobrachella melica *	0																
**42**	* Leptobrachella minima *	14.6	0															
**43**	* Leptobrachella nyx *	11.8	5.7	0														
**44**	* Leptobrachella oshanensis *	15.1	10.4	8.9	0													
**45**	* Leptobrachella pallida *	11.1	14.7	15.2	16.4	0												
**46**	* Leptobrachella picta *	14.4	16.5	14.7	17.6	15.3	0											
**47**	* Leptobrachella pluvialis *	14.2	7.8	6.4	11.1	14.8	15.7	0										
**48–49**	* Leptobrachella pyrrhops *	12.3–12.7	15.6–16.0	14.4–14.8	16.0–16.4	8.3–8.6	16.4–16.8	14.3–14.8	0.3									
**50**	* Leptobrachella sabahmontana *	12.9	15.7	15.1	15.9	13.3	5.0	16.9	15.0–15.4	00								
**51–53**	* Leptobrachella tengchongensis *	14.5	7.8	8.2	8.2	16.7	16.0	10.3	16.8–17.2	16.4	0							
**54–56**	* Leptobrachella ventripunctata *	15.5–15.9	6.8–7.9	5.0–6.0	11.1–12.3	17.6–18.1	15.5–16.4	8.2–8.6	15.2–15.6	14.8–15.6	9.7–10.8	0–0.1						
**57**	* Leptobrachella zhangyapingi *	17.0	11.4	9.5	11.1	17.5	18.1	10.3	16.7–17.1	18.5	9.2	11.1–11.5	0					
**58–61**	* Leptobrachella rowleyae *	6.3	14.7	12.7	15.9	10.8	15.5	13.5	11.2–11.6	13.9	15.0	16.1–16.5	17.1	0				
**62**	Leptobrachium cf. chapaense	26.1	25.5	25.7	27.5	26.2	25.9	25.1	23.8–24.3	27.5	26.3	24.4–24.8	29.1	25.9	0			
**63**	* Pelobates syriacus *	23.5	26.4	24.9	26.2	25.6	21.1	27.9	24.3–24.7	20.8	28.2	25.4–26.9	28.6	24.7	21.3	0		
**64**	* Pelobates varaldii *	23.5	27.4	24.0	25.7	25.8	21.2	27.8	25.5–26.0	21.6	28.1	24.5–25.9	27.2	25.0	23.1	3.6	0	
**65**	* Megophrys major *	27.1	30.5	28.0	26.6	30.3	27.6	28.2	29.0–29.5	27.2	26.0	26.2–27.7	31.3	29.2	27.9	24.3	22.4	0

### Systematics

#### Leptobrachella yunkaiensis

Taxon classificationAnimaliaAnuraMegophryidae

Wang, Li, Lyu & Wang sp. n.

http://zoobank.org/CE563BA1-D6F5-40BE-ADEC-324190B239EA

Figures 3 , 4C1–C3

##### Holotype.

SYS a004665, adult male, collected on 15 April 2016 by Jian Wang (JW hereafter), Zhao-Chi Zeng (ZCZ hereafter), Ying-Yong Wang (YYW hereafter), Zu-Yao Liu (ZYL hereafter), Hai-Long He (HLH hereafter) and Zhi-Tong Lyu (ZTL hereafter) from Dawuling Forest Station (DWL hereafter) (22°16'32.9"N, 111°11'42.87"E; 1600 m a.s.l.), Maoming City, Guangdong Province, China.

##### Paratypes.

Collectors and locality data of paratypes were the same as holotype: adult males, SYS a004664 / CIB107272, SYS a004666–4669 and an adult female SYS a004663, collected on 15 April 2016, the other adult female, SYS a004690, collected on 16 April 2017.

##### Diagnosis.

(1) small size (SVL 25.9–29.3 mm in males, 34.0–35.3 mm in females), (2) dorsal skin shagreened with short skin ridges and raised warts, (3) iris bicolored, coppery orange on upper half and silver on lower half, (4) tympanum distinctly discernible, slightly concave, weakly black supratympanic line present, (5) dorsal surface yellowish-brown grounding, with distinct darker brown markings and rounded spots and scattered with irregular orange patches, (6) flanks with several dark blotches, (7) surface of belly pinkish, with distinct or indistinct light dark brown speckling, (8) supra-axillary, femoral, pectoral and ventrolateral glands distinctly visible, (9) absence of webbing and presence of distinct lateral fringes on fingers, toes with rudimentary webbing and wide lateral fringes, (10) longitudinal ridges under toes not interrupted at the articulations, and (11) dense conical spines present on lateral and ventral surface of tarsus, surface of tibia-tarsal, inner-side surface of shank and surface around cloacal region.

**Figure 3.**
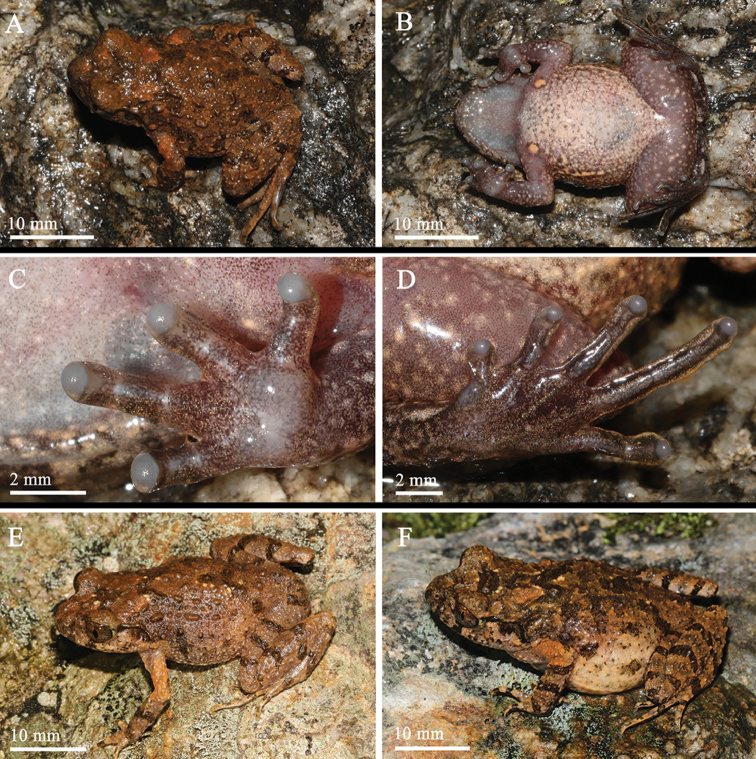
General aspect in life: **A–D**SYS a004665, the male holotype of *Leptobrachellayunkaiensis* sp. n. **E**SYS a004667, the male paratype **F**SYS a004690, the female paratype.

##### Comparisons.

Comparative morphological data of *Leptobrachellayunkaiensis* sp. n. with 66 recognized *Leptobrachella* species were obtained from examination of museum specimens (see Appendix 1) and from the references listed in Table 2. All comparative data were shown in Tables 4, 5.

Compared with the 24 known congeners of the genus *Leptobrachella* occurring south of the Isthmus of Kra, by the presence of supra-axillary and ventrolateral glands, *L.yunkaiensis* sp. n. can be easily distinguished from *L.arayai*, *L.dringi*, *L.fritinniens*, *L.gracilis*, *L.hamidi*, *L.heteropus*, *L.kajangensis*, *L.kecil*, *L.marmorata*, *L.melanoleuca*, *L.maura*, *L.picta*, *L.platycephala*, *L.sabahmontana* and *L.sola*, all of which lacking supra-axillary and ventrolateral glands; and by the significantly larger body size, SVL 25.9–29.3 mm in males, 34.0–35.3 mm in females, *L.yunkaiensis* sp. n. differs from the smaller *L.baluensis* (14.9–15.9 mm in males), *L.brevicrus* (17.1–17.8 mm in males), *L.itiokai* (15.2–16.7 mm in males), *L.juliandringi* (17.0–17.2 mm in males and 18.9–19.1 mm in females), *L.mjobergi* (15.7–19.0 mm in males), *L.natunae* (17.6 mm in male), *L.parva* (15.0–16.9 mm in males and 17.8 mm in female), L.palmata (14.4–16.8 mm in males), *L.serasanae* (16.9 mm in female) and Dring’s (1983)*Leptobrachella* sp. 3 “*baluensis*” (15.0–16.0 mm in males).

**Figure 4.**
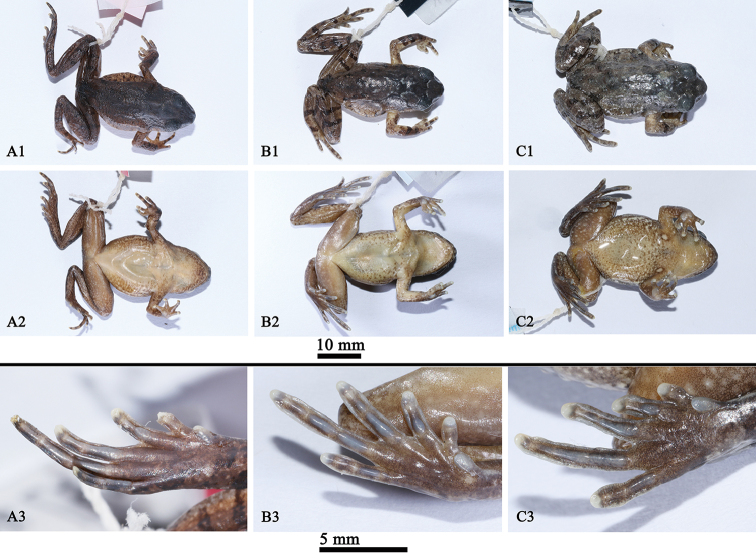
Specimens in preservative: **A1–A3**SYS a002957, the holotype of *Leptobrachellalaui***B1–B3**SYS a005925, the topotype of *L.liui***C1–C3**SYS a004665, the holotype of *L.yunkaiensis* sp. n..

*Leptobrachellayunkaiensis* sp. n. is most similar to *L.laui* and *L.liui*, but it can be distinguished by the larger body sized, SVL 34.0–35.3 mm in females (vs. SVL 28.1 mm in a single female of *L.laui*; SVL 23.0–28.0 mm in females of *L.liui*), presence of short skin ridge and raised warts on dorsum (vs. absent in *L.laui*), black supratympanic line weak (vs. black supratympanic line distinct in *L.liui*), longitudinal ridges under toes not interrupted at the articulations (vs. interrupted in *L.liui*) (Figure 4), belly pinkish with distinct or indistinct speckling (vs. belly creamy white with dark brown dusting on margins in *L.laui*; belly creamy white with dark brown spots on chest and margins in *L.liui*).

From the remaining 40 known congeners (Table 5), with SVL 25.9–29.3 mm in six males, SVL 34.0–35.3 mm in two females in *Leptobrachellayunkaiensis* sp. n., it can be distinguished from the larger *L.eos* (males 33.1–34.7 mm, female 40.7 mm), *L.nahangensis* (male 40.8 mm), *L.pyrrhops* (males 30.8–34.3 mm), *L.sungi* (males 48.3–52.7 mm, females 56.7–58.9 mm) and *L.zhangyapingi* (males 45.8–52.5 mm), and the smaller *L.applebyi* (males 19.6–22.3 mm, females21.7–25.9 mm), *L.melica* (males 19.5–22.7 mm), and *L.pluvialis* (males 21.3–22.3 mm). By having wide fringes on toes, the new species differs from *L.applebyi*, *L.ardens*, *L.crocea*, *L.kalonensis*, *L.lateralis*, *L.maculosa*, *L.macrops*, *L.melica*, *L.minima*, *L.nahangensis*, *L.nyx*, *L.oshanensis*, *L.pallida*, *L.pluvialis*, *L.pyrrhops*, *L.rowleyae*, *L.tadungensis*, *L.tuberosa*, and *L.ventripunctata*, all of which have no lateral fringes on toes; *L.bidoupensis*, *L.bourreti*, *L.fuliginosa*, and *L.sungi*, all of which have weak lateral fringes on toes; *L.botsfordi*, *L.maoershanensis*, *L.pelodytoides*, *L.petrops*, *L.puhoatensis*, and *L.tengchongensis*, all of which have narrow lateral fringes on toes; *L.alpinus*, *L.firthi*, and *L.isos*, all of which have wide lateral fringes only in males. With rudimentary webbing on toes, the new species differs from *L.ardens*, *L.kalonensis*, *L.maculosa*, *L.oshanensis*, *L.pallida*, *L.petrops*, *L.rowleyae*, and *L.tadungensis*, all of which have no webbing on toes; *L.pelodytoides*, *L.sungi*, and *L.tamdil*, all of which have wide webbing on toes. By having black spots on flanks, the new species differs from *L.aerea*, *L.botsfordi*, *L.eos*, *L.firthi*, *L.isos*, *L.pallida*, *L.petrops*, *L.tuberosa*, and *L.zhangyapingi*, all of which have no black spots on flanks. With belly pink with distinct or indistinct speckling, the new species differs from *L.bourreti*, *L.eos*, *L.firthi*, *L.khasiorum*, *L.lateralis*, *L.minima*, *L.nahangensis*, and *L.nokrekensis*, all of which have creamy white belly without patterns; from *L.macrops*, which have greyish-violet with white speckling; from *L.purpura*, which have dull white belly with indistinct grey dusting; and from *L.yingjiangensis*, which have creamy white belly with dark brown flecks on chest and margins. By dorsal skin shagreened with short skin ridges and raised warts, the new species differs from *L.purpura*, *L.yingjiangensis* and *L.tengchongensis*, all of which have shagreened dorsal skin with small tubercles, and from *L.macrops*, which have no skin ridges dorsally.

**Table 4.** Measurements (minimum–maximum (mean ± SD); in mm), and body proportions of *Leptobrachellayunkaiensis* sp. n. from Dawuling Forest Station.

Measurements	Males (n = 6)	Females (n = 2)
**SVL**	25.9–29.3 (27.6 ± 1.4)	34.0–35.3 (34.7 ± 0.9)
**HDL**	9.3–10.3 (9.9 ± 0.4)	12.2–12.6 (12.4 ± 0.2)
**HDW**	9.0–10.0 (9.7 ± 0.4)	12.0–12.2 (12.1 ± 0.1)
**SNT**	3.6–3.8 (3.7 ± 0.1)	4.4–4.7 (4.6 ± 0.2)
**EYE**	3.4–3.7 (3.6 ± 0.1)	3.8–3.9 (3.9 ± 0.1)
**IOD**	2.7–2.9 (2.8 ± 0.1)	3.0–3.2 (3.1 ± 0.1)
**IND**	2.5–2.8 (2.7 ± 0.1)	2.9–3.0 (3.0 ± 0.1)
**TMP**	1.5–1.7 (1.6 ± 0.1)	2.0
**TEY**	0.7–0.8 (0.8 ± 0.1)	1.0
**TIB**	12.2–12.8 (12.5 ± 0.2)	15.0–15.2 (15.1 ± 0.2)
**ML**	5.8–7.3 (6.9 ± 0.6)	7.4–7.8 (7.6 ± 0.2)
**PL**	10.8–12.4 (11.9 ± 0.6)	12.7–12.9 (12.8 ± 0.1)
**LAHL**	12.0–12.6 (12.3 ± 0.2)	14.7–15.0 (14.8 ± 0.2)
**HLL**	37.0–40.3 (38.7 ± 1.2)	47.0–49.5 (48.3 ± 1.8)
**HDL/HDW**	1.01–1.03 (1.02 ± 0.01)	1.02–1.03 (1.02 ± 0.01)
**HDL/SVL**	0.34–0.39 (0.36 ± 0.02)	0.36
**SNT/HDL**	0.36–0.41 (0.38 ± 0.02)	0.37
**SNT/ED**	1.03–1.06 (1.05 ± 0.02)	1.16–1.21 (1.18 ± 0.03)
**EYE/TMP**	2.12–2.40 (2.25 ± 0.13)	1.90–1.95 (1.93 ± 0.04)
**TMP/EYE**	0.42–0.47 (0.45 ± 0.03)	0.51–0.53 (0.52 ± 0.01)
**TEY/TMP**	0.47–0.53 (0.48 ± 0.03)	0.50
**TIB/SVL**	0.43–0.48 (0.45 ± 0.02)	0.43–0.44 (0.44 ± 0.01)
**LAHL/SVL**	0.43–0.47 (0.45 ± 0.02)	0.42–0.43 (0.43 ± 0.01)
**HLL/SVL**	1.33–1.51 (1.41 ± 0.06)	1.38–1.40 (1.39 ± 0.01)
**TIB/HLL**	0.31–0.33 (0.32 ± 0.01)	0.31–0.32 (0.32 ± 0.01)

**Table 5.** Selected diagnostic characters for species described herein and species in the genus *Leptobrachella* occurring north of the Isthmus of Kra (modified from Rowley et al. 2017; Yuan et al. 2017).

Species	Male SVL (mm)	Black spots on flanks	Toes webbing	Fringes on toes	Ventral coloration	Dorsal skin texture
*L.yunkaiensis* sp. n.	25.9–29.3	Yes	Rudimentary	Wide	Belly pink with distinct or indistinct speckling	Shagreened with short skin ridges and raised warts
*L.wuhuangmontis* sp. n.	25.6–30.0	Yes	Rudimentary	Narrow	Greyish white mixed by tiny white and black dots	Rough, scattered with dense conical tubercles
* L. aerea *	25.1–28.9	No	Rudimentary	Wide	Near immaculate creamy white, brown specking on margins	Finely tuberculate
* L. alpinus *	24.0–26.4	Yes	Rudimentary	Wide in males	Creamy-white with dark spots	Relatively smooth, some with small warts
* L. applebyi *	19.6–22.3	Yes	Rudimentary	No	Reddish brown with white speckling	Smooth
* L. ardens *	21.3–24.7	Yes	No	No	Reddish brown with white speckling	Smooth- finely shagreened
* L. bidoupensis *	18.5–25.4	Yes	Rudimentary	Weak	Reddish brown with white speckling	Smooth
* L. botsfordi *	29.1–32.6	No	Rudimentary	Narrow	Reddish brown with white speckling	Shagreened
* L. bourreti *	28.0–36.2	Yes	Rudimentary	Weak	Creamy white	Relatively smooth, some with small warts
* L. crocea *	22.2–27.3	No	Rudimentary	No	Bright orange	Highly tuberculate
* L. eos *	33.1–34.7	No	Rudimentary	Wide	Creamy white	Shagreened
* L. firthi *	26.4–29.2	No	Rudimentary	Wide in males	Creamy white	Shagreened with fine tubercles
* L. fuliginosa *	28.2–30.0	Yes	Rudimentary	Weak	White with brown dusting	Nearly smooth, few tubercles
* L. isos *	23.7–27.9	No	Rudimentary	Wide in males	Creamy white with white dusting on margins	Mostly smooth, females more tuberculate
* L. kalonensis *	25.8–30.6	Yes	No	No	Pale, speckled brown	Smooth
* L. khasiorum *	24.5–27.3	Yes	Rudimentary	Wide	Creamy white	Isolated, scattered tubercles
* L. lateralis *	26.9–28.3	Yes	Rudimentary	No	Creamy white	Roughly granular
* L. laui *	24.8–26.7	Yes	Rudimentary	Wide	Creamy white with dark brown dusting on margins	Round granular tubercles
* L. liui *	23.0-28.7	Yes	Rudimentary	Wide	Creamy white with dark brown spots on chest and margins	Round granular tubercles with glandular folds
* L. macrops *	28.0–29.3	Yes	Rudimentary	No	Greyish-violet with white speckling	Roughly granular with larger tubercles
* L. maculosa *	24.2–26.6	Yes	No	No	Brown, less white speckling	Mostly smooth
* L. maoershanensis *	25.2–30.4	Yes	Rudimentary	Narrow	Creamy white chest and belly with irregular black spots	Longitudinal folds
* L. melica *	19.5–22.7	Yes	Rudimentary	No	Reddish brown with white speckling	Smooth
* L. minima *	25.7–31.4	Yes	Rudimentary	No	Creamy white	Smooth
* L. nahangensis *	40.8	Yes	Rudimentary	No	Creamy white with light specking on throat and chest	Smooth
* L. nokrekensis *	26.0–33.0	Yes	Rudimentary	unknown	Creamy white	Tubercles and longitudinal folds
* L. nyx *	26.7–32.6	Yes	Rudimentary	No	Creamy white with white with brown margins	Rounded tubercles
* L. oshanensis *	26.6–30.7	Yes	No	No	Whitish with no markings or only small, light grey spots	Smooth with few glandular ridges
* L. pallida *	24.5–27.7	No	No	No	Reddish brown with white speckling	Tuberculate
* L. pelodytoides *	27.5–32.3	Yes	Wide	Narrow	Whitish	Small, smooth warts
* L. petrops *	23.6–27.6	No	No	Narrow	Immaculate creamy white	Highly tuberculate
* L. pluvialis *	21.3–22.3	Yes	Rudimentary	No	Dirty white with dark brown marbling	Smooth, flattened tubercles on flanks
* L. puhoatensis *	24.2–28.1	Yes	Rudimentary	Narrow	Reddish brown with white dusting	Longitudinal skin ridges
* L. purpura *	25.0–27.5	Yes	Rudimentary	Wide	Dull white with indistinct grey dusting	Shagreen with small tubercles
* L. pyrrhops *	30.8–34.3	Yes	Rudimentary	No	Reddish brown with white speckling	Slightly shagreened
* L. rowleyae *	23.4–25.4	Yes	No	No	Pinkish milk-white to light brown chest and belly with numerous white speckles	Smooth with numerous tiny tubercles
* L. sungi *	48.3–52.7	No or small	Wide	Weak	White	Granular
* L. tadungensis *	23.3–28.2	Yes	No	No	Reddish brown with white speckling	Smooth
* L. tamdil *	32.3	Yes	Wide	Wide	White	Weakly tuberculate
* L. tengchongensis *	23.9–26.0	Yes	Rudimentary	Narrow	White with dark brown blotches	Shagreened with small tubercles
* L. tuberosa *	24.4–29.5	No	Rudimentary	No	White with small grey spots/streaks	Highly tuberculate
* L. ventripunctata *	25.5–28.0	Yes	Rudimentary	No	Chest and belly with dark brown spots	Longitudinal skin ridges
* L. yingjiangensis *	25.7–27.6	Yes	Rudimentary	Wide	Creamy white with dark brown flecks on chest and margins	Shagreened with small tubercles
* L. zhangyapingi *	45.8–52.5	No	Rudimentary	Wide	Creamy-white with white with brown margins	Mostly smooth with distinct tubercles

##### Description of holotype.

Adult male. Body size small, SVL in 28.7 mm. Head length slightly larger than head width, HDL/HDW 1.03; snout slightly protruding, projecting slightly beyond margin of the lower jaw; nostril equidistance between snout and eye; canthus rostralis gently rounded; loreal region slightly concave; interorbital space flat, larger internarial distance; pineal ocellus absent; vertical pupil; snout length slightly larger than eye diameter, SNT/EYE 1.03; tympanum distinct, rounded, and slightly concave, diameter smaller than that of the eye and larger than tympanum-eye distance, TMP/EYE 0.46 and TEY/TMP 0.47; weakly black supratympanic line present; vomerine teeth absent; vocal sac openings slit-like, located posterolaterally on floor of mouth in close proximity to the margins of the mandible; tongue deeply notched behind; supratympanic ridge distinct, extending from posterior corner of eye to supra-axillary gland; tubercles present on supratympanic ridge.

Tips of fingers rounded, slightly swollen; relative finger lengths I = II = IV < III; nuptial pad absent; subarticular tubercles absent; a large, rounded inner palmar tubercle distinctly separated from small, round outer palmar tubercle; absence of webbing and presence of distinct lateral fringes on fingers. Tips of toes like fingers; relative toe length I < II < V < III < IV; subarticular tubercles absent; distinct dermal ridges present under the 3^rd^ to 5^th^ toes; large, oval inner metatarsal tubercle present, outer metatarsal tubercle absent; toes webbing rudimentary; wide lateral fringes present on all toes. Tibia 43% of snout-vent length; tibiotarsal articulation reaches to middle of eye; heels just meeting each other when thighs are appressed at right angles with respect to body.

Skin on dorsum shagreened and scattered with fine, round tubercles; short skin ridges and raised warts on dorsum surface present; ventral skin smooth; pectoral gland and femoral gland large, oval; pectoral glands greater than tips of fingers and femoral glands; femoral gland situated on posteroventral surface of thigh, closer to knee than to vent; supra-axillary gland raised. Ventrolateral gland distinctly visible, forming an incomplete line. Dense conical spines on lateral and ventral surface of tarsus, surface of tibia-tarsal, inner-side surface of shank and surface around cloacal region present.

##### Measurements of holotype

**(in mm).**SVL 28.7, HDL 10.3, HDW 10.0, SNT 3.8, EYE 3.7, IOD 2.9, IND 2.8, TMP 1.7, TEY 0.8, TIB 12.4, ML 7.2, PL 12.1, LAHL 12.3, HLL 38.3.

##### Coloration of holotype in life.

Dorsal surface orange-brown with distinct dark brown blotches edged distinct light orange pigmentation. A dark brown triangular pattern between eyes, connected to the dark brown W-shaped marking between axillae. Tympanum black. Orange-brown tubercles present on dorsum of body and limb, those on flanks much distinct and dense; anterior upper lip with distinct blackish brown patches; transverse dark brown bars on dorsal surface of limbs; indistinct dark brown blotches on flanks from groin to axilla; elbow and upper arms without dark bars but with distinct coppery orange coloration; fingers and toes with indistinct dark brown blotches.

Surface of throat creamy white and scattered with small whitish dots; belly pinkish and scattered with small brown speckling; ventral surface of thighs pinkish and scattered with small light orange-brown spots. Supra-axillary coppery orange; femoral, pectoral and ventrolateral glands whitish orange. Iris bicolored, coppery orange on upper half and silver on lower half.

##### Coloration of holotype in preservative.

Dorsum of body and hindlimbs dark brown while dorsum of forelimbs yellowish brown; transverse bars on limbs become more distinct, dark brown patterns, markings and spots on back become indistinct. Ventral surface of body yellowish brown, with brown marbling on sides and chest. Orange supra-axillary, femoral, pectoral and ventrolateral glands fade to greyish white (Figure 4C1–C3).

##### Sexual dimorphism.

Females with a larger body size than males, SVL 34.0–35.3 mm (34.7 ± 0.9) (vs. SVL 25.9–29.3 mm (27.6 ± 1.4) in males); presence of a single vocal sac in males (vs. absent in females); dense conical spines on lateral and ventral surface of tarsus, surface of tibia-tarsal, inner-side surface of shank, surface of thighs and surface around cloacal region distinct in males, and barely visible in females.

##### Variations.

All paratypes match the overall characters of the holotype except that: the heels just meeting each other when thighs are appressed at right angles with respect to body, tibiotarsal articulation reaches to middle of eye in holotype SYS a004665 (vs. tibiotarsal articulation reaches to anterior corner of eye in SYS a004666, reaches the posterior corner of eye in SYS a004669). Surface of belly scattered with distinctly dark brown speckling in holotype (vs. such speckling indistinct in female paratypes SYS a004663, 4690. Tympanum black in the holotype (vs. tympanum black grounding with orange speckling in SYS a004667–4668). Distinct black spots present on dorsum in the female paraype SYS a004690 (Figure 3).

##### Etymology.

The specific epithet, *yunkaiensis*, is in reference to the type locality, DWL of Guangdong, China located in the Yunkai Mountains Range. For the common name, we suggest “Yunkai Mountain’s Leaf Litter Toad”, and Chinese name “Yun Kai Zhang Tu Chan (云开掌突蟾)”.

##### Distribution and habits.

**Figure 5.**
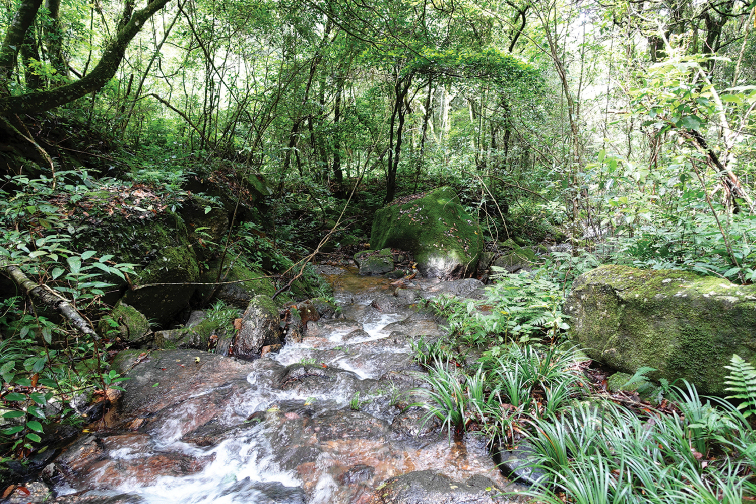
The habitat of *Leptobrachellayunkaiensis* sp. n. in Dawuling Forest Station of Guangdong Province.

Currently, *Leptobrachellayunkaiensis* sp. n. is known only from its type locality DWL of Guangdong Province (Figure 1). The new species was found along a clear-water rocky stream (ca. 2–3 m in width and ca. 20–30 cm in depth) and small nearby seeps in well-preserved montane evergreen broadleaf forest (1600 m a.s.l.) (Figure 5). During April and June, males were found calling mainly hidden under leaf litter, and some were found calling perching on the rocks or under rocks by the side of the stream. Females collected on April bear pure white oocytes.

#### Leptobrachella wuhuangmontis

Taxon classificationAnimaliaAnuraMegophryidae

Wang, Yang & Wang sp. n.

http://zoobank.org/C87E92AA-081E-480B-839C-27CED127F6CA

Figures 6 , 7

##### Holotype.

SYS a003486, adult male, collected on 29 March 2015 by JW, ZTL, YYW and ZYL from Mt. Wuhuang (MWH hereafter) (22°08'30.77"N, 109°24'43.90"E; 500 m a.s.l.), Pubei County, Qinzhou City, Guangxi Province, China.

##### Paratypes.

Adult males SYS a000578, 581 and an adult female SYS a000580, collected on 28 April 2009 by Jian-Huan Yang (JHY hereafter) and Run-Lin Li (RLL hereafter), adult males SYS a003487–3489, 3505–3506, SYS a003500 / CIB107274 and adult females SYS a003485, 3499, 3504,, collected from 29–30 March 2015 by JW, ZTL, YYW and ZYL, all from the same locality as the holotype.

##### Diagnosis.

(1) small size (SVL 25.6–30.0 mm in males, 33.0–36.0 mm in females), (2) dorsal surface rough with skin ridges and dense conical tubercles, (3) iris bicolored, coppery yellow on upper half and silver on lower half, (4) tympanum distinctly discernible, slightly concave, dark brown, distinct black supratympanic line present, (5) dorsal surface greyish purple background with dark brown markings and scattered with orange-yellow blotches and white speckling, (6) distinct dark blotches on flanks, (7) ventral surface greyish white mixed by tiny white and black dots, (8) lateral fringes on fingers absent, (9) toes with narrow lateral fringes and rudimentary webbing, (10) longitudinal ridges under toes not interrupted at the articulations, and (11) dense conical spines on lateral and ventral surface of tarsus, dorsal surface of tibia-tarsal and surface of inner-side shank and surface around cloacal region.

**Figure 6.**
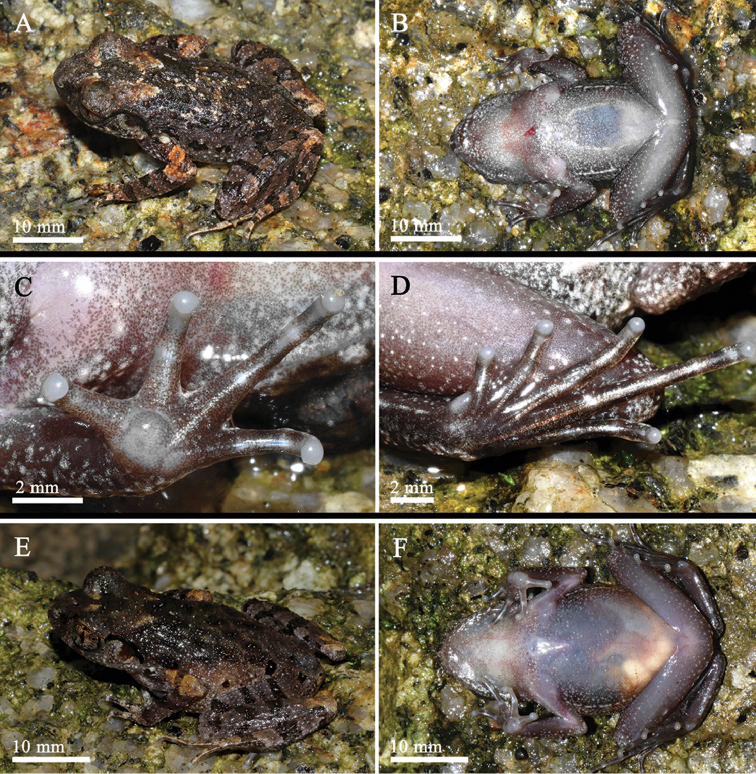
General aspect in life of SYS a003486 (**A–D**), the male holotype of *Leptobrachellawuhuangmontis* sp. n. and the female paratype SYS a003499 (**E, F**).

**Figure 7.**
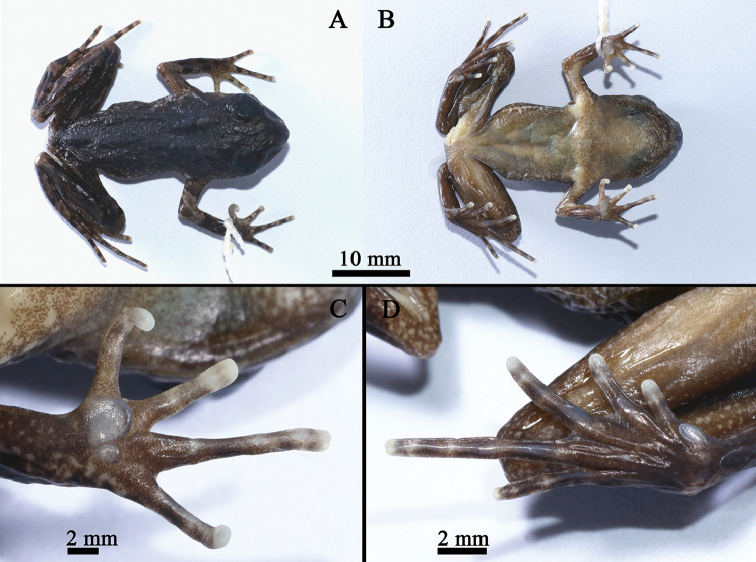
The holotype of *Leptobrachellawuhuangmontis* sp. n., SYS a003486 in preservative.

##### Comparisons.

Comparative morphological data of *Leptobrachellawuhuangmontis* sp. n. with the 66 recognized *Leptobrachella* species were obtained from examination of museum specimens (see Appendix 1) and from the references listed in Table 2. All comparative data were shown in Tables 4, 5, 6.

Compared with the 24 known congeners of the genus *Leptobrachella* occurring south of the Isthmus of Kra, by the presence of supra-axillary and ventrolateral glands, *L.wuhuangmontis* sp. n. can be easily distinguished from *L.arayai*, *L.dringi*, *L.fritinniens*, *L.gracilis*, *L.hamidi*, *L.heteropus*, *L.kajangensis*, *L.kecil*, *L.marmorata*, *L.melanoleuca*, *L.maura*, *L.picta*, *L.platycephala*, *L.sabahmontana*, and *L.sola*, all of which lacking supra-axillary and ventrolateral glands; and by the significantly larger body size, SVL 25.6–30.0 mm in males, 33.0–36.0 mm in females, *L.wuhuangmontis* sp. n. differs from the smaller *L.baluensis* (14.9–15.9 mm in males), *L.brevicrus* (17.1–17.8 mm in males), *L.itiokai* (15.2–16.7 mm in males), *L.juliandringi* (17.0–17.2 mm in males and 18.9–19.1 mm in females), *L.mjobergi* (15.7–19.0 mm in males), *L.natunae* (17.6 mm in male), *L.parva* (15.0–16.9 mm in males and 17.8 mm in female), L.palmata (14.4–16.8 mm in males), *L.serasanae* (16.9 mm in female) and Dring’s (1983)*Leptobrachella* sp. 3 “*baluensis*” (15.0–16.0 mm in males).

*Leptobrachellawuhuangmontis* sp. n. significantly differs from *L.yunkaiensns* sp. n. by a large genetic divergence (*p*=10.2–11.1%), lateral fringes on toes narrow (vs. wide), black supratympanic line distinct (vs. weak), dorsal surface of body rough and scattered with dense conical tubercles (vs. shagreened with short skin ridges and raised warts), belly greyish white mixed by tiny white and black dots (vs. belly pink with distinct or indistinct speckling).

From the rest 42 known congeners (Table 5), with SVL 25.6–30.0 mm in nine males and 33.0–36.0 mm in four females, *Leptobrachellawuhuangmontis* sp. n. differs from the larger *L.bourreti* (females 42.0–45.0 mm), *L.eos* (males 33.1–34.7 mm, female 40.7 mm), *L.lateralis* (female 36.6 mm), *L.nahangensis* (male 40.8 mm), *L.nyx* (females 37.0–41.0 mm), *L.sungi* (males 48.3–52.7 mm, females 56.7–58.9 mm), *L.tamdil* (male 32.3 mm) and *L.zhangyapingi* (males 45.8–52.5 mm); and from the smaller *L.aerea* (females 28.8–28.9 mm), *L.ardens* (female 24.5 mm), *L.alpinus* (females 32.1–32.5 mm in), *L.applebyi* (males 19.6–20.8 mm, female 21.7 mm), *L.bidoupensis* (males 18.5–25.4 mm), *L.botsfordi* (females 30.0–31.8 mm), *L.kalonensis* (females 28.9–30.6 mm), *L.laui* (female 28.1 mm), *L.liui* (females 23.0–28.0 mm), *L.maculosa* (female 27.0 mm), *L.maoershanensis* (female 29.1 mm), *L.melica* (males 19.5–22.7 mm), *L.oshanensis* (female 31.6 mm), *L.pluvialis* (males 21.0–22.0 mm), *L.puhoatensis* (females 27.3–31.5 mm), *L.rowleyae* (females 27.0–27.8 mm), *L.tadungensis* (female 32.1 mm), and *L.tengchongensis* (females 28.9–28.9 mm). Having head longer than wide in the new species (vs. head wider than long in *L.bourreti*, *L.khasiorum*, *L.lateralis* and *L.sungi*, and head width equal to or wider than long in *L.nokrekensis*). By having narrow fringes on toes, the new species differs from *L.applebyi*, *L.ardens*, *L.crocea*, *L.kalonensis*, *L.lateralis*, *L.maculosa*, *L.macrops*, *L.melica*, *L.minima*, *L.nahangensis*, *L.nyx*, *L.oshanensis*, *L.pallida*, *L.pluvialis*, *L.pyrrhops*, *L.rowleyae*, *L.tadungensis*, *L.tuberosa*, and *L.ventripunctata*, all of which have no lateral fringes on toes; *L.bidoupensis*, *L.bourreti*, *L.fuliginosa*, and *L.sungi*, all of which have weak lateral fringes on toes; *L.alpinus*, *L.firthi* and *L.isos*, all of which have wide lateral fringes only in males; *L.aerea*, *L.eos*, *L.khasiorum*, *L.laui*, *L.liui*, *L.purpura*, *L.tamdil*, *L.yingjiangensis*, and *L.zhangyaping*, all of which have wide lateral fringes both in males and females. By having rudimentary webbing on toes, the new species differs from *L.ardens*, *L.kalonensis*, *L.maculosa*, *L.oshanensis*, *L.pallida*, *L.petrops*, *L.rowleyae*, and *L.tadungensis*, all of which have no webbing on toes; *L.pelodytoides*, *L.sungi*, and *L.tamdil*, all of which have wide webbing on toes. By having black spots on flanks, the new species differs from *L.aerea*, *L.botsfordi*, *L.eos*, *L.firthi*, *L.isos*, *L.pallida*, *L.petrops*, *L.tuberosa*, and *L.zhangyapingi*, all of which have no black spots on flanks. By having rough dorsal skin with skin ridges and dense conical tubercles, the new species differs from *L.applebyi*, *L.bidoupensis*, *L.kalonensis*, *L.melica*, *L.minima*, *L.nahangensis*, and *L.tadumgensis*, all of which have smooth dorsal skin, and from *L.purpura*, *L.tengchongensis*, and *L.yingjiangensis*, all of which have shagreened dorsal skin with small tubercles.

**Table 6.** Measurements (minimum–maximum (mean ± SD); in mm), and body proportions of *Leptobrachellawuhuangmontis* sp. n. from Mt. Wuhuang.

Measurements	Males (n = 9)	Females (n = 4)
**SVL**	25.6–30.0 (28.5 ±1.5)	33.0–36.0 (34.9 ± 1.4)
**HDL**	10.5–11.5 (10.9 ± 0.4)	12.4–12.6 (12.5 ± 0.1)
**HDW**	10.0–11.2 (10.5 ± 0.4)	12.1–12.3 (12.2 ± 0.1)
**SNT**	3.6–4.4 (4.1 ± 0. 2)	4.6–4.7 (4.6 ± 0.1)
**EYE**	3.5–4.4 (4.0 ± 0.3)	4.5–4.6 (4.6 ± 0.1)
**IOD**	2.8–3.0 (2.9 ± 0.1)	3.1–3.3 (3.2 ± 0.1)
**IND**	2.9–3.2 (3.1 ± 0.1)	3.2–3.4 (3.3 ± 0.1))
**TMP**	2.1–2.6 (2.4 ± 0.1)	2.6–2.8 (2.7 ± 0.1)
**TEY**	0.7–0.9 (0.8 ± 0.1)	0.8–0.9 (0.9 ± 0.1)
**TIB**	12.5–13.6 (13.3 ± 0.3)	15.0–16.3 (15.7 ± 0.5)
**ML**	7.0–8.0 (7.6 ± 0.3)	8.0–9.2 (8.5 ± 0.5)
**PL**	11.7–13.0 (12.5 ± 0.5)	13.9–14.8 (14.4 ± 0.4)
**LAHL**	14.2–16.0 (14.9 ± 0.6)	15.8–17.0 (16.4 ± 0.5)
**HLL**	38.8–44.9 (42.8 ± 1.9)	47.5–54.0 (51.2 ± 2.9)
**HDL/HDW**	1.03–1.06 (1.04 ± 0.01)	1.02–1.03 (1.03 ± 0.01)
**HDL/SVL**	0.36–0.41 (0.38 ± 0.02)	0.35–0.38 (0.36 ± 0.01)
**SNT/HDL**	0.34–0.40 (0.38 ± 0.02)	0.37 (0.37 ± 0)
**SNT/ED**	1.00–1.08 (1.03 ± 0.03)	1.00–1.02 (1.01 ± 0.01)
**EYE/TMP**	1.56–1.79 (1.68 ± 0.06)	1.64–1.73 (1.69 ± 0.04)
**TMP/EYE**	0.58–0.64 (0.60 ± 0.02)	0.58–0.61 (0.59 ± 0.01)
**TEY/TMP**	0.28–0.38 (0.33 ± 0.04)	0.30–0.33 (0.31 ± 0.02)
**TIB/SVL**	0.45–0.50 (0.47 ± 0.02)	0.44–0.47 (0.45 ± 0.01)
**LAHL/SVL**	0.50–0.55 (0.52 ± 0.02)	0.46–0.49 (0.47 ± 0.02)
**HLL/SVL**	1.45–1.54 (1.50 ± 0.03)	1.41–1.52 (1.47 ± 0.05)
**TIB/HLL**	0.29–0.33 (0.31 ± 0.01)	0.29–0.32 (0.31 ± 0.01)

##### Description of holotype.

Adult male. Body size small, SVL in 30.0 mm. Head slightly longer than wide, HDL/HDW 1.04; snout rounded in dorsal view, nostril rounded, closer to tip of snout than to eye; canthus rostralis distinct; lores slightly concave; eye large, diameter equal to snout length, in 4.3 mm; tympanum distinct, rounded and slightly concave, its diameter significantly shorter than eye, TMP/EYE 0.56; distinct black supratympanic line present; vomerine teeth absent; vocal sac opening slit-like; tongue deeply notched behind; supratympanic ridge distinct, running from eye towards supra-axillary gland with raised tubercles.

Tips of fingers rounded, slightly swollen; relative finger lengths I = II < IV < III; nuptial pad absent; subarticular tubercles absent; a large, rounded inner palmar tubercle distinctly separated from small, round outer palmar tubercle; finger webbing absent and lateral fringes absent. Tips of toes like fingers; relative toe length I < II < V < III < IV; subarticular tubercles absent; dermal ridges undeveloped but present under the 3^rd^ to 5^th^ toes; large, oval inner metatarsal tubercle present, outer metatarsal tubercle absent; toes webbing rudimentary; narrow lateral fringes present on all toes. Tibia 45% of snout-vent length; tibiotarsal articulation reaches to middle of eye; heels just meeting each other when thighs are appressed at right angles with respect to body.

Skin on dorsum body and limbs rough with skin ridges and dense conical tubercles, ventral skin smooth; pectoral gland and femoral gland large, oval, slightly elevated; femoral gland situated on posterovertral surface of thigh, closer to knee than to vent; supra-axillary gland raised. Ventrolateral gland distinct, forming an incomplete line. Dense conical spines present on surface of lateral and ventral tarsus, surface of tibia-tarsal, inner-side surface of shank and surface around cloacal region.

##### Measurements of holotype

**(in mm).**SVL 30.0, HDL 10.9, HDW 10.5, SNT 4.3, EYE 4.3, IOD 2.9, IND 3.0, TMP 2.4, TEY 0.8, TIB 13.5, ML 7.8, PL 13.0, LAHL 15.4, HLL 44.9.

##### Coloration of holotype in life.

Dorsal surface greyish purple with distinct dark brown markings and scattered with yellow blotches; distinct small white speckling present on edges of dark markings. A distinct dark brown triangle pattern between eyes, connected to the incomplete W-shaped dark brown marking between axillae. Tubercles on dorsum of body and limbs brown, those on lower flanks somewhat whitish; anterior upper lip with distinct blackish brown patches; transverse dark brown bars on dorsal surface of limbs; distinct dark brown blotches on flanks from groin to axilla; elbow and upper arms coppery orange and with distinct dark bars; fingers and toes with distinct dark brown blotches.

Ventral surface greyish-white mixed with tiny white and black dots. Supra-axillary, femoral, and ventrolateral glands white, pectoral gland greyish white as the color of ventral surface. Iris bicolored, coppery yellow on upper half and silver on lower half.

##### Coloration of holotype in preservative.

Dorsal of body dark with greyish white dots on flanks, while dorsal of limbs dark brown, transverse bars on dorsal of forelimbs become more distinct, and indistinct on dorsal of hindlimbs, dark brown patterns, markings and spots on back become indistinct. Ventral surface light yellow with brown speckling. Supra-axillary, femoral, ventrolateral and pectoral glands light yellow (Figure 7).

##### Sexual dimorphism.

Females with a larger body size than males, SVL 33.0–36.0 mm (34.9 ± 1.4) (vs. SVL 25.6–30.0 mm (28.5 ±1.5) in males); presence of a single vocal sac in males (vs. absent in females); dense conical spines on lateral and ventral surface of tarsus, surface of tibia-tarsal, inner-side surface of shank and surface around cloacal region distinct in males (vs. barely visible in females); pectoral gland and femoral gland large, oval, slightly elevated in males (vs. indistinct in females).

##### Variations.

All paratypes match the overall characters of the holotype except that: tibiotarsal articulation reaches to posterior corner of eye in female paratypes SYS a003499, 3504 and reaches to anterior corner of eye in male paratypes SYS a003487 and SYSa 003500 / CIB 107274; pectoral gland large, oval, slightly elevated in all individuals in life, and become indistinct in preservation. Yellow blotches and white speckling present on dorsum in the holotype (vs. indistinct in the female paratype SYS a003499). Elbow and upper arms coppery orange and with distinct dark bars in the holotype (vs. elbow and upper arms light orange, dark bars indistinct in the male paratypes SYS a003488, 3505 and the female paratype SYS a003499) (Figure 6).

##### Etymology.

The specific epithet, *wuhuangmontis*, is in reference to the type locality, Mt. Wuhuang of Guangxi Province, China. For the common name, we suggest “Mt. Wuhuang’s Leaf Litter Toad”, and for the Chinese name “Wu Huang Shan Zhang Tu Chan (五皇山掌突蟾)”.

##### Distribution and habits.

**Figure 8.**
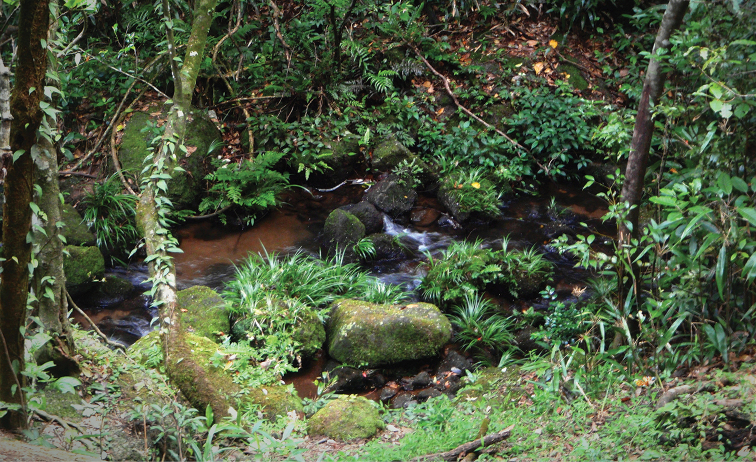
The habitat of *Leptobrachellawuhuangmontis* sp. n. in Mt. Wuhuang of Guangxi Province.

Currently, *Leptobrachellawuhuangmontis* sp. n. is only known from its type locality MWH from Guangxi Province of China (Figure 1). The new species was found along a clear-water rocky streams and small steep rocky streams in well-preserved montane evergreen broadleaf forest (500 m a.s.l.) (Figure 8). During field surveys in March, males were found calling exposed on the rocks or hiding in the rock seams; gravid female collected on March and April bear pure white oocytes.

## Discussion

Studies of the taxonomy and phylogeny of *Leptobrachella* are difficult to perform because of the morphological conservativeness and very similar characters (for example, the coloration and the texture of skin) in different environments, which may cause misidentifications (Ohler et al. 2010; Sung et al. 2014). With the evidence of both morphological and phylogenetic analyses, 15 cryptic species of the genus *Leptobrachella* have been discovered and described since 2010 (Frost 2017; Rowley et al. 2016, 2017; Yang et al. 2016; Yuan et al. 2017). With the description of *L.yunkaiensis* sp. n. and *L.wuhuangmontis* sp. n. based on an taxonomical approach, the number of the genus *Leptobrachella* herein is increased to 68, indicating the underestimated diversity.

During our examination, it was observed that the dense tiny conical spines present on the surface of the lateral and ventral aspects of the tarsus, surface of tibia-tarsal, the inner surface of the shank and surface around cloacal region (distinct in males and barely visible in females) in the two new *Leptobrachella* species described in this study are also present in examined specimens of *L.alpinus*, *L.laui*, *L.liui*, and *L.tengchongensis* as well as in other cryptic taxa (Wang et al. unpublished data). Thus, this neglected morphological character may be common among congeners of the genus *Leptobrachella*, and further morphological studies are needed to study this in more detail.

Mt. Wuhuang of Guangxi Province in southern China is known for the extraordinarily high biodiversity, with some new national records discovered in recent years, for example, the national records of *Opisthotropismaculosa* and *Sphenomorphustonkinensis* from Mt. Wuhuang were recorded (Wang et al. 2013; Yang et al. 2011). Except for the new species (*Leptobrachellayunkaiensis* sp. n.) described in this study, several new species of amphibians and reptiles have been discovered from Dawuling Forest Station during field surveys in the last two years (Wang et al. unpublished data; Wang et al. 2018; Lyu et al. 2018), which suggests a high herpetofaunal biodiversity of Dawuling Forest Station localized in western Guangdong Province, China. Recently, these areas have been subjected to tourism development; thus, conservation strategies and measures for references and enforcements are urgently needed.

## Supplementary Material

XML Treatment for Leptobrachella yunkaiensis

XML Treatment for Leptobrachella wuhuangmontis

## Appendix 1

**Specimens examined**

*Leptobrachellaalpinus* (n = 6): China: Yunnan Province: Jingdong County: Mt. Wuliang: CIB 24353 (Holotype), CIB 24354; SYS a 003927.

*Leptobrachellalaui* (n = 26): China: Hong Kong: SYS a002057 (Holotype), SYS a002058; China: Guangdong Province: Shenzhen City: SYSa 001505–1507, 1515–1521, 3471–3472, 5644–5645.

*Leptobrachellaliui* (n = 18): China: Fujian Province: Mt. Wuyi: CIB 24355 (Holotype), CIB 24356, SYS a001571–1578, 1595–1599, 2478–2479, 5925–5826.

*Leptobrachellatengchongensis* (n = 6): China: Yunnan Province: Baoshan City: Mt. Gaoligong: SYS a004600 (Holotype), 4596–4599, 4601–4602.
